# Small-Pox in Philadelpia and Elsewhere

**Published:** 1871-10

**Authors:** 


SMALL-POX IN PHILADELPHIA AND ELSEWHERE.—Last weeks the deaths from small-pox, in this city, numbered seventy-four—nearly half of them adults. The public is alarmed, and with reason, for the ravages of this dreaded disease in Paris and London the last two years have been such as to recall, though somewhat faintly, the terrible pictures of its devastation, which are contained in medical treatises, before the days of inoculation and vaccination.

Active measures have been taken by the public authorities to extend the facilities of vaccination, and to impress upon the public mind the importance of this simple precautionary measure. Except among the lowest class of population there is no objection to its reception, and all that is needed is energetic action on the part of our Board of Health—not a very energetic body, we regret to say—to secure the city entire immunity from the scourge.

At tnis time the importance of *revaccination* cannot too strongly be insisted upon. We have published, within the last few months, several able articles from foreign writers upon this point, foreseeing the approach of the disease. Through mistaken views upon it, we regret that some eminent medical men have committed themselves to the opinion that revaccination is needless, or indifferent. Such views are contrary to known facts, and fraught with injurious consequences to the community. They are next in character to the folly of opposing vaccination because once in a million cases it conveys syphilis into the system.—*Phila. Med. Reporter.*

